# The complete chloroplast genome sequence of *Dendrobium thyrsiflorum* (Orchidaceae) and its phylogenetic analysis

**DOI:** 10.1080/23802359.2019.1691947

**Published:** 2019-11-18

**Authors:** Bin Zhu, Xi Luo, Zuomin Gao, Qun Feng, Lijuan Hu, Qingbei Weng

**Affiliations:** School of Life Sciences, Guizhou Normal University, Guiyang, People’s Republic of China

**Keywords:** *Dendrobium thyrsiflorum*, chloroplast genome, PacBio platform, phylogenetic analysis

## Abstract

*Dendrobium thyrsiflorum* Rchb.f., a native species to China, is widely used as an important garden flower and a traditional Chinese medicine. Herein, the complete chloroplast (cp) genome of *D. thyrsiflorum* was deciphered by high-throughput sequencing. The cp genome exhibited a typical quadripartite cycle of 151,686 bp in length, comprising of a pair of inverted repeats (IRa and IRb) of 26,293 bp which were intersected by a large single copy (LSC) region of 84,749 bp and a small single copy (SSC) region of 14,351 bp. A total of 126 genes were *de novo* assembled in this cp genome, including 78 protein genes, 40 tRNA genes and 8 rRNA genes. Among these genes, 86 genes (22 tRNAs and 64 coding genes) were single copy, the rest were two-copy genes, and the average of GC content of the whole genome is 37.55%. Phylogenetic trees showed that the *D. thyrsiflorum* was closely related to *D. devonianum*. This study provides molecular information for future evolution, genetic and molecular biology studies of *Dendrobium*.

The *Dendrobium* species, occupying the largest family members of Orchidaceae, have been widely used as traditional Chinese medicine (Wojcikowski and Gobe 2014; Zhang et al. 2018) and popular garden flowers (Gao et al. 2018). *Dendrobium thyrsiflorum* Rchb. f. which is distributed in South China has been reported effective in treatment of various chronic disorders and anti-blood coagulant (Bhattacharyya et al. 2015). Due to the over-exploitation of wild resources and narrow distribution, the wild populations of *D. thyrsiflorum* are severely threatened. Furthermore, absence of genome information makes it difficult to conserve this species using the modern breeding techniques. Here, we *de novo* assembled the complete chloroplast (cp) genome of *D. thyrsiflorum* through the combination of PacBio Sequel and Illumina HiSeq platform. This study provides molecular information for future conservation, evolution, and molecular biology studies of *D. thyrsiflorum*.

The samples of *D. thyrsiflorum* were collected from Jingdong Yi Autonomous County, Puer City, China (100°22′12″E, 23°56′26″N) and transplanted to the green house (accession number: SH-12) in Guizhou Normal University. Then cp DNA was extracted from 5 g fresh leaves using CTAB method. After quality assessment, two DNA libraries with insert size of 20 kb and 400 bp fragments were prepared and sequenced by PacBio platform and Illumina HiSeq 4000, respectively. Illumina reads were *de novo* assembled scaffolds using SOAPdenovo v2.04 with the default options. Then, PacBio reads were error-corrected with Illumina shot reads using PacBioToCA (Koren et al. 2012) to reduce the insertion/deletion of single base. These corrected PacBio reads were used to gap-fill the scaffolds by PBgelly (English et al. 2012) to generate a circular cp genome. Finally, the well-annotated cp genome was deposited in GenBank (accession number: MN413199).

The complete cp genome sequence of *D. pendulum* is 151,686 bp in length, including a large single-copy (LSC) region of 84,749 bp, a small single-copy (SSC) region of 14,351 bp, and two spaced inverted repeat (IRa and IRb) regions of 26,293 bp. The cp genome contains 126 genes (88 unique): 78 protein-coding genes, 40 tRNA genes, and 8 rRNA genes. Among these genes, 58 protein-coding genes and 23 tRNA genes are distributed in LSC region, whereas six protein-coding genes and one tRNA genes are located in SSC region. The average of GC content of the whole genome is 37.55%. Because of harboring GC-rich rRNA genes, the IRs region shows the highest GC content of 43.43% among three different regions (35.10% for LSC and 30.44% for SSC).

To detect the phylogenetic relation of *D. pendulum* with other *Dendrobium* species, coding genes sequences of 28 cp genomes of *Dendrobium* genus were used to construct phylogenetic trees by Neighbor-joining method using MEGA7 (Kumar et al. 2016). To enhance the tree stability, 1000 replicates were tested during the bootstrap analysis. Phylogenetic trees showed that *D. pendulum* was closely related to *D. devonianum* with a node value of 99. The complete cp genome sequence of *D. pendulum* can be of great benefit to further study on its population conservation, genetics and molecular breeding (Figure 1).

**Figure 1.**
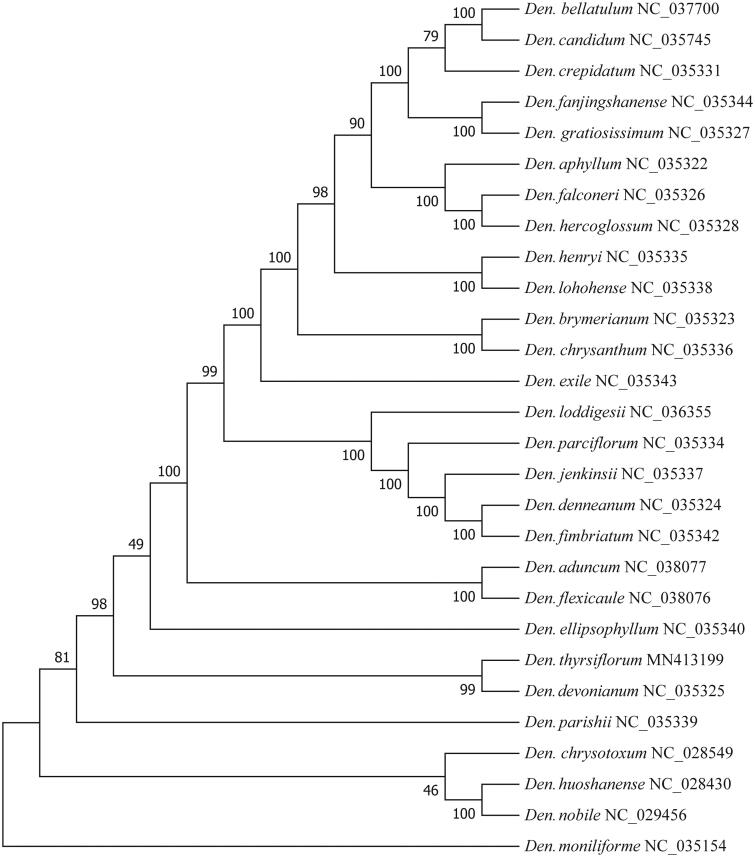
Phylogenetic trees of 28 *Dendrobium species*, based on protein-coding genes in the cp genome. The trees were generated by Neighbor-joining method with 1000 replicates.
